# Imaging of synchronous multifocal renal masses: a case report

**DOI:** 10.3389/fmed.2026.1906732

**Published:** 2026-08-14

**Authors:** Tong Zhang, Siyu Chen, Yaxin Li, Di Xu, Xiaoqi Huang

**Affiliations:** 1 Department of Radiology, Jinhua People's Hospital, Jinhua, China; 2 Department of Radiology, Affiliated Hospital of Yan'an University, Yan'an, China

**Keywords:** case report, CT urography, ganglioneuroma, kidney neoplasms, PEComa, renal oncocytoma, ultrasonography

## Abstract

**Background:**

Synchronous multifocal renal masses (SMRMs) are defined as the coexistence of two or more histologically distinct tumors within the same kidney. Such combinations are rare and frequently mimic multifocal renal cell carcinoma (RCC) or other malignancies, complicating diagnosis and management. The synchronous occurrence of renal oncocytoma (RO), perivascular epithelioid cell tumor (PEComa), and ganglioneuroma (GN) in a single kidney is exceptionally rare.

**Case presentation:**

A 79-year-old woman was admitted with a 1-year history of an incidentally detected right renal mass on ultrasonography. The mass was initially discovered on ultrasonography during a routine health screening; the patient declined further evaluation at that time due to financial constraints and the absence of symptoms. She had no flank pain, gross hematuria, fever, or weight loss. Physical examination revealed right costovertebral angle tenderness on percussion, without palpable masses. Laboratory tests, including renal function and urinalysis, were unremarkable. Renal ultrasonography demonstrated a heterogeneous hypoechoic mass in the upper–mid pole and a slightly hyperechoic mass in the lower pole of the right kidney; both showed internal strip-like and peripheral annular blood flow on color Doppler. CT urography revealed three right-sided lesions with distinct imaging characteristics involving the upper pole, renal hilum, and lower pole. Chest CT was performed for staging and showed no evidence of pulmonary metastasis. Laparoscopic radical right nephrectomy was performed after multidisciplinary discussion. Histopathology confirmed RO in the upper pole, PEComa in the lower pole, and GN at the renal hilum, supported by immunohistochemical staining. The patient was counseled regarding the potential for a hereditary syndrome given the multifocal nature of her tumors; formal genetic testing was recommended but the patient declined. The postoperative course was uneventful. At 1-month follow-up, ultrasonography showed no recurrence and compensatory hypertrophy of the contralateral kidney, with preserved renal function.

**Conclusion:**

This case highlights a unique combination of three benign tumors of distinct lineages within a single kidney. Although histopathology remains the gold standard, cross-sectional imaging is critical for characterizing SMRMs and differentiating them from multifocal malignancy. Genetic counseling should be considered in patients presenting with multifocal renal tumors of diverse histology.

## Introduction

Synchronous multifocal renal masses (SMRMs) are defined as two or more renal tumors with different histologic types occurring simultaneously or within a short interval in the same kidney (1). The true incidence of SMRMs remains unknown, as available data are limited to case reports and small series. A systematic review by Zhang et al. (2) estimated that multiple primary renal tumors account for approximately 5%−7% of all renal tumors, and among these, synchronous tumors with distinct histologies represent a distinct subset. This clinical entity presents a significant diagnostic challenge, as multifocal lesions are often presumed to represent multifocal renal cell carcinoma (RCC) or intrarenal metastasis from a single primary, potentially leading to unnecessary radical surgery when nephron-sparing approaches might be feasible (3).

Although the coexistence of common renal tumors, such as renal cell carcinoma (RCC) and angiomyolipoma (AML), is occasionally observed, the combination of renal oncocytoma (RO), perivascular epithelioid cell tumor (PEComa), and ganglioneuroma (GN)—arising from epithelial, mesenchymal, and neural tissues, respectively—is extremely rare. Each of these tumors has distinct embryologic origins and histologic features. RO arises from the intercalated cells of the collecting duct and is the most common benign solid renal tumor, accounting for approximately 3%−7% of all adult renal tumors (4). PEComa is a mesenchymal tumor defined by the presence of perivascular epithelioid cells that co-express melanocytic (HMB-45, Melan-A) and myogenic (smooth muscle actin) markers (5); renal PEComas are uncommon, and up to 20% of cases may be associated with tuberous sclerosis complex (TSC) (6). GN is a benign neurogenic tumor arising from sympathetic ganglion cells, most commonly occurring in the retroperitoneum and adrenal medulla, and is exceptionally rare in the renal hilum (7). To our knowledge, this specific triad has not been previously reported.

Most patients with SMRMs lack typical clinical manifestations such as flank pain, hematuria, or palpable abdominal mass and are often diagnosed incidentally on routine imaging (1). Only a minority present with mild flank discomfort due to mass effect from larger lesions. Vasudev et al. (8) reported that up to 55% of renal tumors are detected incidentally on imaging. The asymptomatic nature of these tumors increases the difficulty of early clinical recognition. Furthermore, the differential diagnosis of SMRMs is broad, encompassing multifocal RCC, hereditary syndromes such as Birt-Hogg-Dubé syndrome and tuberous sclerosis complex, and various benign entities (9, 10). The pathogenetic mechanisms underlying their synchronous occurrence remain incompletely understood (11–13).

Herein, we report a case of SMRMs comprising RO, PEComa, and GN within a single kidney, confirmed by postoperative histopathology and immunohistochemistry. We describe the clinical, imaging, and pathologic findings in accordance with the CARE guidelines (14) and discuss the differential diagnosis and management considerations to improve awareness of this rare condition among clinicians and radiologists.

## Case presentation

### Patient information

A 79-year-old woman presented with a right renal mass incidentally detected on screening ultrasonography 1 year prior. She declined further evaluation at that time due to financial constraints and absence of symptoms. She remained asymptomatic during the subsequent year, until a follow-up ultrasound at a local facility suggested malignancy, prompting referral to our institution (Table 1).

**Table 1:** Timeline of clinical events.

Time point	Event
1 year prior to admission	Incidental detection of right renal mass on routine health screening ultrasonography at local facility; patient declined further evaluation
1 month prior to admission	Follow-up ultrasonography at local facility suggested possible malignancy; referral to our institution
Day 0 (admission)	Physical examination, laboratory investigations, renal ultrasonography, and CT urography performed
Day 1	Chest CT performed for staging; no pulmonary metastasis identified
Day 2	Multidisciplinary team (MDT) discussion; decision for laparoscopic radical right nephrectomy
Day 3	Laparoscopic radical right nephrectomy performed under general anesthesia
Postoperative days 1–7	Recovery; wound pain management, gradual return of bowel function
Postoperative day 8	Discharged in good condition
1 month postoperative	Follow-up ultrasonography: no recurrence, compensatory hypertrophy of left kidney; preserved renal function

The patient reported occasional urinary frequency and urgency but denied dysuria. Her medical history was significant for hypertension (poorly controlled), lacunar cerebral infarction, and bilateral cataracts. She had no history of diabetes, malignancy, or smoking. Her family history was non-contributory, with no known renal tumors, hereditary cancer syndromes, or tuberous sclerosis complex among first- or second-degree relatives.

### Clinical findings

On admission, the patient was alert and in good general condition. Blood pressure was 148/103 mmHg and temperature was 36.5 °C. Urologic examination revealed tenderness to percussion at the right costovertebral angle. No skin lesions (fibrofolliculomas, angiofibromas, or facial papules) suggestive of BHD syndrome were noted.

### Diagnostic assessment

#### Laboratory investigations

Serum creatinine was 120.0 μmol/L (reference: 44–133 μmol/L), blood urea nitrogen was 8.3 mmol/L (reference: 2.9–8.2 mmol/L), and uric acid was 112 μmol/L (reference: 155–357 μmol/L). Estimated glomerular filtration rate (eGFR) was 42.6 ml/min/1.73 m^2^. Urinalysis was negative for protein, red blood cells, and leukocytes. Serum tumor markers, including carcinoembryonic antigen (CEA), carbohydrate antigen 125 (CA-125), and carbohydrate antigen 19-9 (CA19-9), were all within normal limits.

#### Imaging findings

##### Ultrasonography

Renal ultrasonography demonstrated a heterogeneous hypoechoic mass measuring approximately 7.5 × 5.9 cm in the middle–upper pole of the right kidney. The lesion was partly intrapelvic and partly exophytic, with a well-defined but irregular contour and inhomogeneous internal echogenicity. Color Doppler flow imaging (CDFI) showed annular peripheral vascular signals and punctate/strip-like internal flow signals.

**Figure 1:**
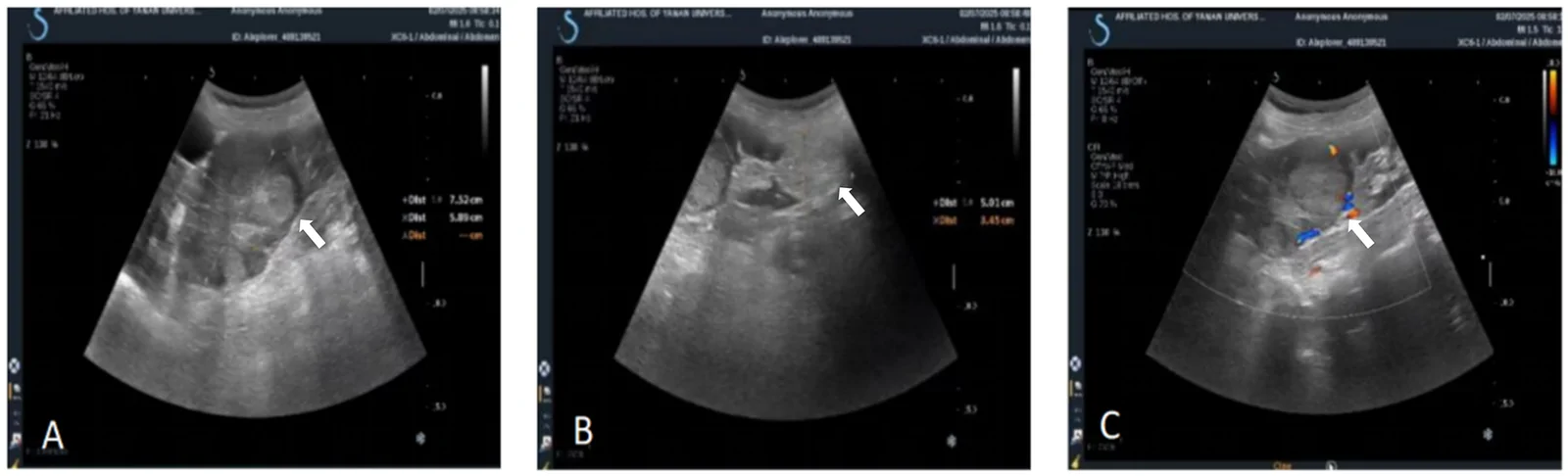
Renal ultrasonography. **(A)** Gray-scale ultrasonography shows a heterogeneous hypoechoic mass in the upper–mid pole of the right kidney (white arrows). **(B)** A second, slightly hyperechoic exophytic mass is seen in the lower pole of the right kidney (white arrows). **(C)** Color Doppler flow imaging demonstrates peripheral annular vascularity with punctate/strip-like intralesional flow (white arrows).

A second lesion in the lower pole of the right kidney appeared as a slightly hyperechoic, exophytic mass measuring approximately 5.0 × 3.4 cm, with a smooth outline but heterogeneous internal echotexture. A simple cyst was noted in the left kidney (Figure 1).

##### CT urography

**Figure 2:**
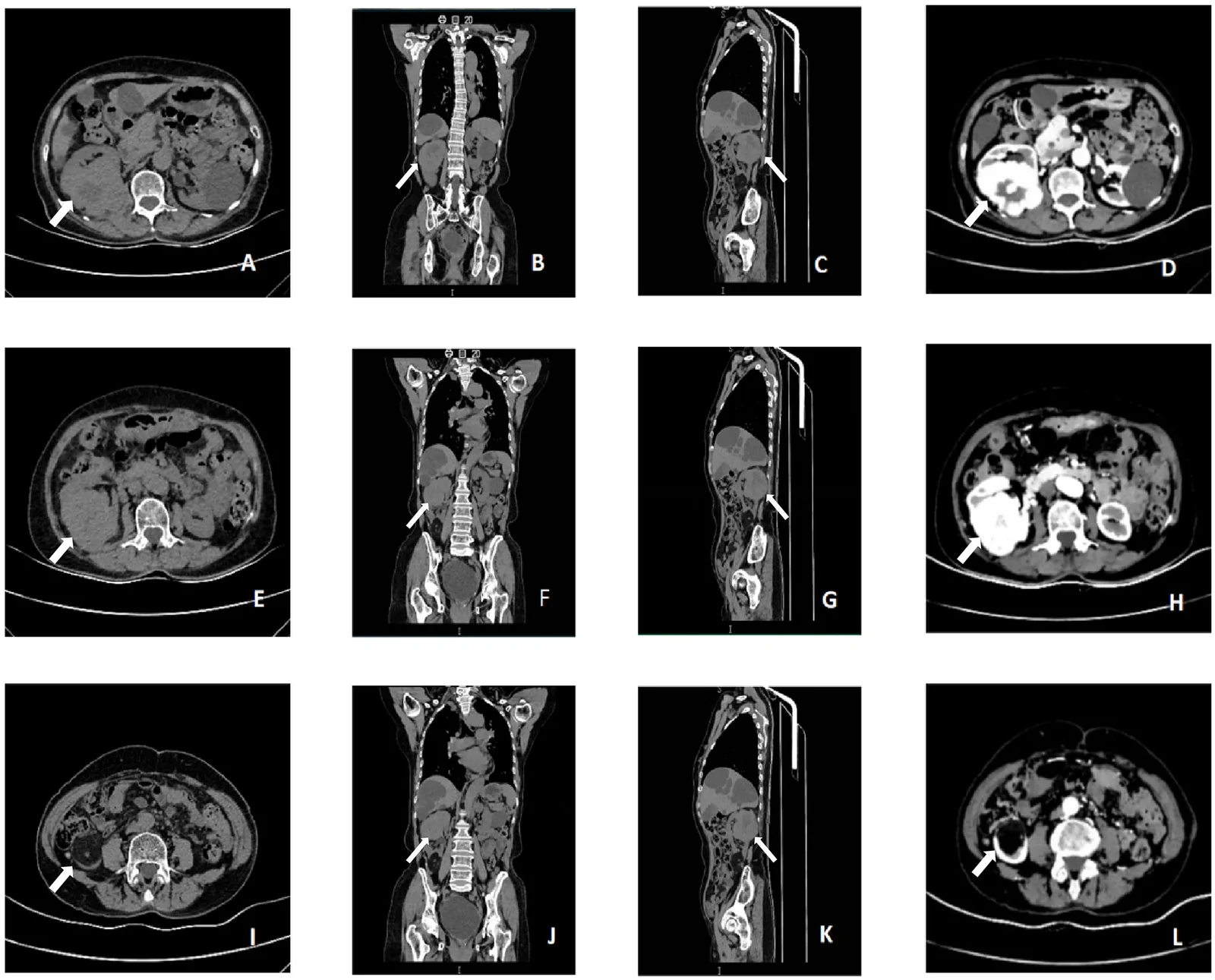
CT urography (CTU) of the right kidney demonstrating three discrete lesions with different enhancement patterns. **(A–D)** Upper-pole mass: axial, coronal, and sagittal noncontrast images and the corticomedullary (arterial) phase show an irregular mixed-attenuation lesion with marked heterogeneous enhancement (white arrows). **(E–H)** Hilar mass: corresponding multiplanar noncontrast and corticomedullary-phase images demonstrate a mixed-attenuation lesion with avid enhancement (white arrows). **(I–L)** Lower-pole lesion: multiplanar images show a round low-attenuation mass with focal enhancement in the corticomedullary phase and areas of cystic necrosis also visible (white arrows).

CTU revealed abnormal morphology of the right kidney with three discrete lesions (Figure 2):

**1. Upper pole lesion:** An irregular, mixed-attenuation mass (72 × 56 mm) involving the renal pelvis. Non-contrast CT showed heterogeneous soft-tissue density. After contrast administration, the lesion demonstrated marked heterogeneous enhancement during the corticomedullary (arterial) phase with decreased enhancement in the nephrographic and delayed phases.

**2. Hilar lesion:** A mixed-attenuation mass (57 × 46 mm) at the right renal hilum, with obvious enhancement on contrast-enhanced images. The lesion was not detected on the initial ultrasound examination, which may be attributed to its location within the renal sinus fat or to ill-defined margins against the upper and lower pole lesions.

**3. Lower pole lesion:** A round, low-attenuation lesion (31 × 28 mm) in the lower pole. On contrast-enhanced CT, part of the lesion showed distinct enhancement, and areas of cystic necrosis also visible.

In the excretory phase, a focal filling defect was observed within the right renal pelvis. The overall imaging impression was of multifocal right renal solid tumors, likely RCC.

Note on Measurement Discrepancy: The lower pole lesion measured 5.0 × 3.4 cm on ultrasonography but only 31 × 28 mm on CT, likely reflecting differences in imaging planes and the inclusion of cystic areas on ultrasound that were excluded from the solid-enhancing CT measurement.

### Chest CT

Chest CT was performed for staging purposes given the large tumor size (7.5 cm) with suspicious imaging features, and revealed no evidence of pulmonary metastasis, mediastinal lymphadenopathy, or pleural effusion (15).

### Diagnostic reasoning

Preoperative imaging raised concern for multifocal RCC. However, several features suggested a more complex diagnosis: (1) the three lesions exhibited markedly different imaging characteristics—notably heterogeneous enhancement with a possible central scar, avid enhancement, and cystic components—whereas multifocal RCC typically shows similar enhancement patterns across all lesions; (2) the absence of clinical symptoms despite the large tumor burden favored indolent or benign pathology; and (3) the patient's advanced age and lack of traditional RCC risk factors further supported this possibility.

### Therapeutic intervention

After multidisciplinary discussion and consideration of the patient's age, comorbidities, lesion size, and multifocal distribution, laparoscopic radical right nephrectomy was performed under general anesthesia. Radical nephrectomy was chosen over partial nephrectomy due to the multifocal distribution of three lesions, the large size of the dominant lesion involving the renal pelvis, and the preoperative suspicion of malignancy. The risks and benefits were discussed with the patient and her family, and informed consent was obtained. The kidney and perirenal fat were mobilized, and the renal artery and vein were ligated. The kidney was removed en bloc with the masses. There were no intraoperative complications. Postoperatively, the patient received intravenous fluids, prophylactic antibiotics, and supportive care.

### Follow-up and outcomes

The patient had an uneventful postoperative recovery and was discharged on postoperative day 8. At 1-month follow-up, renal ultrasonography showed no evidence of local recurrence or new lesions, with compensatory hypertrophy of the contralateral kidney and stable renal function. The patient was advised to undergo regular follow-up with renal ultrasonography and CT at 6-month intervals, including chest imaging. Given the presence of three histologically distinct tumors, the patient was referred for genetic counseling; however, she declined testing due to financial constraints. She was informed of the potential hereditary implications and advised to notify first-degree relatives.

#### Pathologic findings

**Gross pathology:** The specimen contained three macroscopically distinct masses.

**Histopathology:**

**1. Upper-pole tumor (RO):** Tumor cells were polygonal with abundant eosinophilic granular cytoplasm and centrally located round nuclei with visible nucleoli. No definite mitotic figures were identified. The cells were arranged in acinar patterns, and a central fibrous stellate scar was present, consistent with renal oncocytoma (RO).

**2. Lower-pole tumor (PEComa):** Composed of epithelioid and spindle cells with rich vasculature. Immunohistochemistry (IHC) was positive for HMB-45 and smooth muscle actin (SMA), consistent with PEComa. Based on the Folpe classification system for PEComas (5), this tumor was classified as having uncertain malignant potential, as it met one worrisome feature (size >5 cm on ultrasonography) but lacked other high-risk features including high nuclear grade, necrosis, vascular invasion, and significant mitotic activity. According to the PEC-PRO prognostic score proposed by Gantzer et al. (16), which incorporates surgical margin status, necrosis, and sex, this tumor would be classified as low risk (score = 0: negative margins, no necrosis, female sex).

**3. Hilar tumor (GN):** Composed of mature Schwann cells, ganglion cells, and nerve fibers, consistent with Ganglioneuroma (GN).

##### Immunohistochemistry

**Figure 3:**
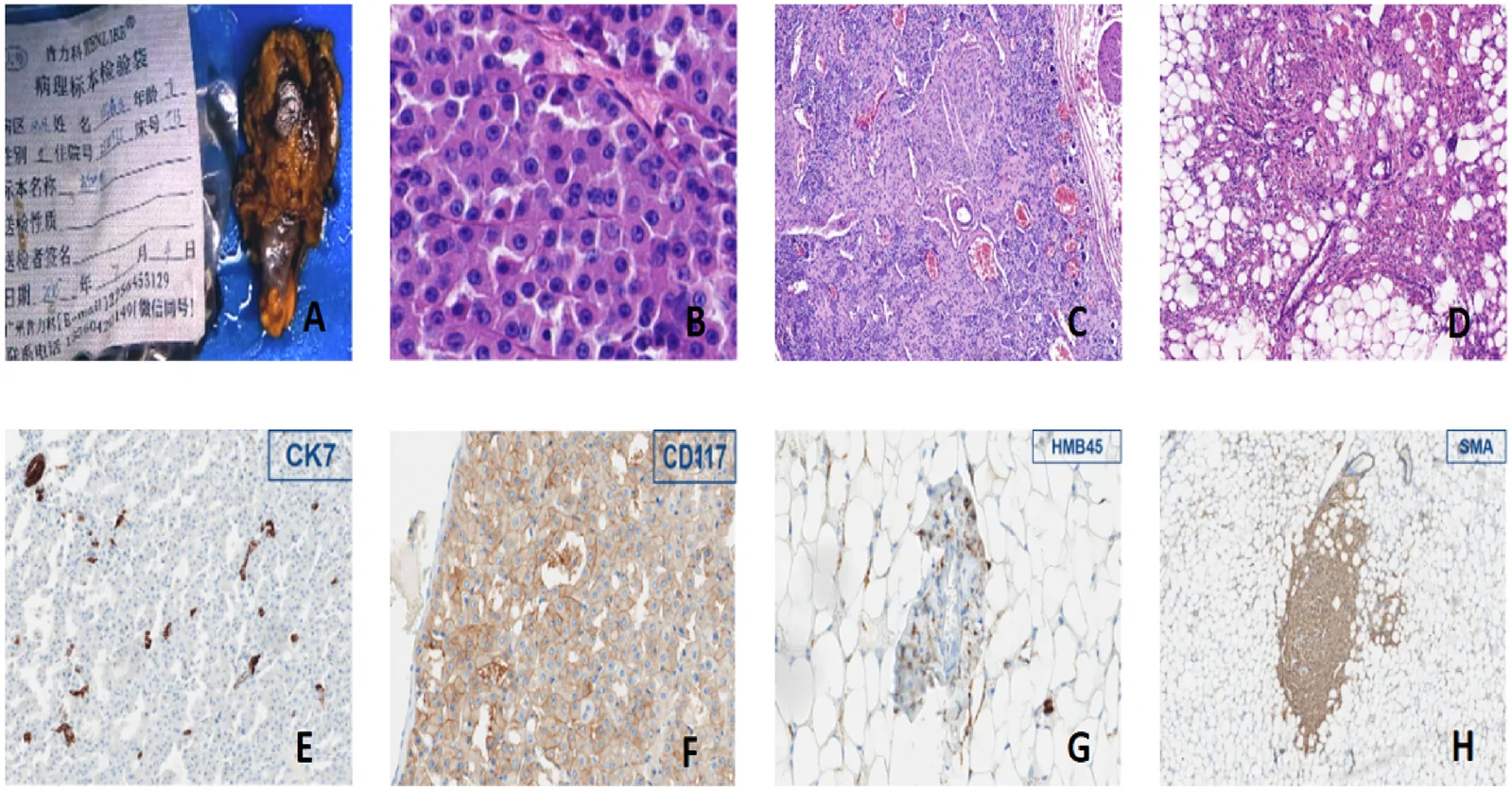
Gross, histopathological, and immunohistochemical findings. **(A)** Gross specimen following radical nephrectomy shows three macroscopically distinct masses located in the upper pole and lower pole. **(B)** Upper-pole tumor: sheets/nests of oncocytic cells with abundant eosinophilic granular cytoplasm and centrally located round nuclei, consistent with renal oncocytoma [hematoxylin and eosin (H&E), × 400]. **(C)** Hilar tumor: mature ganglion cells admixed with Schwann cells/nerve fibers, consistent with ganglioneuroma (H&E, × 100). **(D)** Lower-pole tumor: epithelioid and spindle cells with prominent vasculature, consistent with PEComa (H&E, × 400). **(E, F)** Immunohistochemistry of the upper-pole tumor shows CK7 positivity **(E)** and CD117 positivity **(F)**. **(G, H)** Immunohistochemistry of the lower-pole tumor shows HMB-45 positivity **(G)** and smooth muscle actin (SMA) positivity **(H)**.

Immunohistochemical staining supported these diagnoses. The upper-pole tumor showed positivity for CK7 and CD117. The lower-pole tumor showed positivity for HMB-45 and SMA (Figure 3).

#### Final diagnosis

Based on the clinical, imaging, and pathologic findings, the final diagnosis was synchronous multifocal renal masses of the right kidney, consisting of RO (upper pole), PEComa (lower pole), and GN (renal hilum).

### Patient perspective

The patient expressed satisfaction with the surgical outcome and relief that the tumors were confirmed to be benign. She reported that her initial decision to delay evaluation for 1 year was primarily driven by financial constraints and the absence of symptoms. In retrospect, she acknowledged that earlier evaluation might have allowed for more surgical options, including possible nephron-sparing approaches. The patient expressed gratitude for the care received and a willingness to comply with follow-up recommendations.

## Discussion

### Clinical and epidemiologic considerations

SMRMs are extremely rare. The combination of RO, PEComa, and GN within a single kidney, as in this case, is exceptional (1). These three tumors differ markedly in embryologic origin, histologic features, and biologic behavior; yet their synchronous occurrence in one kidney illustrates a unique example of multifocal benign neoplasia.

Most reported SMRMs occur in middle-aged patients (2), with no substantial sex predilection and without classical risk factors for RCC such as smoking, obesity, or long-standing hypertension. In contrast to aggressive renal malignancies, RO, PEComa, and GN are characterized by indolent growth. Patients often lack the classic triad of flank pain, hematuria, and palpable mass and are instead diagnosed incidentally during routine ultrasonography or CT. Only when lesions exceed approximately 5 cm in diameter and compress perirenal tissues or the collecting system do patients develop dull flank discomfort or mild obstructive urinary symptoms. This asymptomatic nature increases the difficulty of early clinical recognitio n (8).

The one-year delay in our case reflects socioeconomic barriers to timely medical evaluation in elderly patients in rural China, including limited healthcare access, financial constraints, and absence of symptoms (17). This highlights the importance of patient education regarding incidentally detected renal masses.

#### Imaging evaluation and differential diagnosis

Imaging is the first-line modality for detecting and characterizing SMRMs, whereas histopathology and immunohistochemistry are the gold standard for definitive diagnosis.

##### Ultrasonography

Ultrasonography serves as an initial screening tool. In our case, it detected the upper- and lower-pole lesions but failed to identify the hilar GN, likely due to its echogenicity resembling that of surrounding renal sinus fat—a known limitation for renal sinus lesions. The RO appears as a well-circumscribed, iso- to hypoechoic mass with limited peripheral flow (18), the PEComa as a homogeneous hypoechoic lesion with poor vascular signals (19), and the GN tends to show low echogenicity with posterior enhancement and minimal blood flow (20). These findings underscore the limited ability of ultrasonography to characterize internal composition, necessitating CT or MRI for definitive evaluation.

##### CT

CT characterizes lesion density and enhancement patterns. RO generally appears isoattenuating on unenhanced CT and demonstrates a pattern of progressive or sustained enhancement with a non-enhancing central stellate scar (21). PEComa appears hypo- or isoattenuating and shows marked arterial-phase hyperenhancement with washout on later phases, without necrosis or cystic change (22). GN often presents as a low-attenuation mass (10–30 HU) that exhibits mild, gradual enhancement with slightly increased attenuation on delayed phase (7); linear or streaky low-attenuation areas corresponding to myxoid degeneration may be seen.

##### MRI

MRI further delineates tissue composition. RO typically shows isointense signal on T1-weighted images and slightly hypointense signal on T2-weighted images, with the central scar appearing hypointense (23). PEComa is hypointense to isointense on T1-weighted images and hyperintense on T2-weighted images (24), with enhancement patterns similar to CT. GN appears hypointense on T1-weighted images and markedly hyperintense on T2-weighted images (25); myxoid areas are even more hyperintense, and enhancement is mild and progressive.

The main differential diagnoses include:

**1. Multicentric RCC** (especially multifocal clear cell RCC): Clear cell RCC commonly demonstrates rapid wash-in and wash-out of contrast and tends to show similar enhancement behavior among multifocal lesions.Our three lesions had markedly different patterns (homogeneous with scar, heterogeneous with fat, progressive delayed), atypical for RCC (26).

**2. Renal angiomyolipoma (AML) and its variants:** Classic AML is easily recognized by macroscopic fat. However, fat-poor AML can closely resemble PEComa. Both are hypervascular and may contain small amounts of fat. Immunohistochemistry is crucial: PEComa typically shows strong HMB-45 and Melan-A positivity (27), whereas AML often demonstrates focal or negative HMB-45 staining.

**3. Hereditary renal tumor syndromes:** The synchronous occurrence of three histologically distinct tumors raises concern for an underlying hereditary syndrome. BHD syndrome is characterized by multifocal renal tumors of mixed histology, cutaneous fibrofolliculomas, and pulmonary cysts (9). TSC is associated with AMLs, PEComas, and other hamartomatous lesions (6). Our patient lacked the characteristic cutaneous and pulmonary manifestations of either syndrome; however, the absence of overt syndromic features does not exclude a genetic predisposition in the setting of multifocal tumors of diverse histology.

### Pathogenesis and pathologic correlation

From an embryologic perspective, RO, PEComa, and GN arise from distinct cell lineages: distal tubular epithelial cells, perivascular epithelioid cells, and sympathetic ganglion cells, respectively. Histologically, RO is characterized by oncocytic cells with a central stellate scar; PEComa consists of epithelioid and spindle cells that are positive for HMB-45 and SMA (27); and GN contains mature ganglion cells, Schwann cells, and nerve fibers (28).

The mechanism underlying the synchronous occurrence of these three benign tumors in the same kidney remains unclear. Several hypotheses have been proposed, including multifocal differentiation abnormalities within the renal microenvironment (13), shared dysregulation of signaling pathways (11), or an unrecognized genetic predisposition. These possibilities suggest that a common pathogenetic mechanism may contribute to this rare tumor combination.

First, shared dysregulation of the mTOR pathway is a compelling candidate. PEComas commonly harbor TSC1/2 mutations that lead to constitutive mTOR complex 1 (mTORC1) activation (6, 11). A subset of renal oncocytomas also exhibits MTOR mutations or copy number alterations (12, 29), and ganglioneuromas may show upregulation of the PI3K/AKT/mTOR axis (30). The convergence of these three tumor types on the same signaling pathway raises the possibility that a common upstream event could predispose to multifocal benign neoplasia. Second, local microenvironmental factors—such as chronic inflammation, oxidative stress, or a “field effect”—or the presence of a multipotent renal progenitor capable of divergent differentiation could give rise to histologically distinct tumors within the same kidney (13). Furthermore, regarding unrecognized genetic susceptibility, the synchronous occurrence of three histologically distinct tumors within a single kidney strongly suggests an underlying hereditary or syndromic predisposition; however, the majority of MPRT cases still lack an identifiable genetic cause. In a cohort study of 93 patients with multiple primary renal tumors, only 7.5% were found to carry pathogenic germline variants (31).

### Disease progression and treatment decision-making

The management of SMRMs requires careful consideration of multiple factors, including the number, size, and location of lesions; the patient's age, comorbidities, and renal function; and the preoperative suspicion of malignancy.

For SMRMs, nephron-sparing surgery (partial nephrectomy) is optimal when feasible, preserving renal function (32). In our case, the decision for radical nephrectomy was based on several factors: (1) the multifocal distribution of three lesions across the upper pole, renal hilum, and lower pole; (2) the large size of the dominant upper pole lesion (7.5 cm) involving the renal pelvis; (3) the hilar location of the GN, which would make partial nephrectomy with adequate margins extremely challenging; and (4) the need to achieve complete tumor resection given the uncertain malignant potential of the PEComa. We acknowledge that preoperative percutaneous biopsy could have been performed to clarify the histology of the dominant lesion, potentially altering the surgical strategy. However, given the multifocal nature of the disease with three distinct lesions, sampling error was a significant concern, and biopsy of all three lesions was not clinically practical.

For future similar cases, a more individualized “hybrid” surgical strategy could be considered: combining partial nephrectomy for accessible lesions (33, 34), active surveillance for small lesions highly suggestive of benignity (e.g., typical AML or RO), and percutaneous biopsy for atypical or high-risk lesions to refine preoperative diagnosis and reduce the likelihood of unnecessary radical nephrectomy.

## Limitations

This case report has several limitations. First, the patient declined formal genetic testing, so the possibility of an underlying hereditary syndrome cannot be definitively excluded. Second, preoperative percutaneous biopsy was not performed, which may have influenced the surgical strategy if benign histology had been confirmed. Third, although three-dimensional volume-rendered CT reconstruction was attempted, the soft-tissue contrast was insufficient to clearly delineate the renal parenchyma and the spatial relationships among the three lesions.

## Conclusion

This case reports a unique combination of three benign tumors—RO, PEComa, and GN—of distinct lineages within a single kidney. Cross-sectional imaging is critical for detecting and characterizing SMRMs, although definitive diagnosis relies on histopathology and immunohistochemistry. In patients with multifocal renal tumors of diverse histology, genetic counseling should be considered. Nephron-sparing strategies should be explored when technically feasible, and preoperative biopsy may help refine surgical planning. Accumulation of additional cases and further molecular investigations are needed to clarify the pathogenesis and long-term outcomes of such multifocal benign renal tumor combinations.

## Data Availability

The raw data supporting the conclusions of this article will be made available by the authors, without undue reservation.
